# Clinical signs of trachoma are prevalent among Solomon Islanders who have no persistent markers of prior infection with *Chlamydia trachomatis*

**DOI:** 10.12688/wellcomeopenres.13423.2

**Published:** 2018-08-10

**Authors:** Robert Butcher, Oliver Sokana, Kelvin Jack, Leslie Sui, Charles Russell, Anna Last, Diana L. Martin, Matthew J. Burton, Anthony W. Solomon, David C.W. Mabey, Chrissy h. Roberts

**Affiliations:** 1 Clinical Research Department, London School of Hygiene & Tropical Medicine, London, UK; 2 Eye Department, Solomon Islands Ministry of Health and Medical Services, Honiara, Solomon Islands; 3 Primary Care Department, Lata Hospital, Lata, Solomon Islands; 4 Bellona Rural Health Centre, Bellona, Solomon Islands; 5 Division of Parasitic Diseases and Malaria, US Centers for Disease Control and Prevention, Atlanta, GA, USA

**Keywords:** Trachoma, ocular Chlamydia trachomatis, ddPCR, anti-Pgp3 antibodies, trachomatous scarring, Solomon Islands

## Abstract

**Background:** The low population prevalence of trachomatous trichiasis and high prevalence of trachomatous inflammation–follicular (TF) provide contradictory estimates of the magnitude of the public health threat from trachoma in the Solomon Islands. Improved characterisation of the biology of trachoma in the region may support policy makers as they decide what interventions are required. Here, age-specific profiles of anti-Pgp3 antibodies and conjunctival scarring were examined to determine whether there is evidence of ongoing transmission and pathology from ocular *Chlamydia trachomatis *( *Ct*) **infection.

**Methods: **A total of 1511 individuals aged ≥1 year were enrolled from randomly selected households in 13 villages in which >10% of children aged 1–9 years had TF prior to a single round of azithromycin mass drug administration undertaken six months previously. Blood was collected to be screened for antibodies to the *Ct* antigen Pgp3. Tarsal conjunctival photographs were collected for analysis of scarring severity.

**Results: **Anti-Pgp3 seropositivity was 18% in 1–9 year olds, sharply increasing around the age of sexual debut to reach 69% in those over 25 years. Anti-Pgp3 seropositivity did not increase significantly between the ages of 1–9 years and was not associated with TF (p=0.581) or scarring in children (p=0.472). Conjunctival scars were visible in 13.1% of photographs. Mild (p<0.0001) but not severe (p=0.149) scars increased in prevalence with age.

**Conclusions:** Neither conjunctival scars nor lymphoid follicles were associated with antibodies to *Ct,* suggesting that they are unlikely to be a direct result of ocular *Ct *infection *. *Clinical signs of trachoma were prevalent in this population but were not indicative of the underlying rates of *Ct* infection. The current World Health Organization guidelines for trachoma elimination indicated that this population should receive intervention with mass distribution of antibiotics, but the data presented here suggest that this may not have been appropriate.

## Introduction

Trachoma is responsible for approximately 1.9 million cases of visual impairment or blindness globally ^1^. International partners have committed to elimination of trachoma as a public health problem by the year 2020 and the global elimination strategy is guided by the clinical signs trachomatous trichiasis (TT) and trachomatous inflammation–follicular (TF). The World Health Organization (WHO) recommends at least three years of mass drug administration (MDA) with azithromycin in districts with ≥10% TF prevalence in 1–9 year-olds to treat the causative agent *Chlamydia trachomatis* ( *Ct*) ^2^.

A 2013 population-based prevalence survey (PBPS) covering two provinces (Temotu and Rennell & Bellona) of the Solomon Islands showed that the proportion of 1–9-year-old children with TF was moderately high (26.1%) ^3^. In response to this and in accordance with WHO guidelines, MDA took place throughout the Solomon Islands in 2014 and the national program administered approximately 24,000 doses of azithromycin (achieving coverage of approximately 80% in Rennell & Bellona, and 85% in Temotu). Data from the 2013 PBPS suggested that whilst TF was prevalent, TT (0.1% of those ≥15 years), trachomatous inflammation—intense (TI; 0.2% of 1–9-year-olds), and ocular infection with *Ct* (1.3% of 1–9 year-olds) were all rare ^3^. Our recent survey of Kiritimati Island, Kiribati ^4^, used the same tools and estimated more typically-matched values of TF and *Ct* infection prevalence (among children) at 28% and 24%, respectively. We therefore questioned the underlying biology of the TF signs that were observed in the Solomon Islands.

We hypothesised that the clinical signs in the Solomon Islands were not consistent with repeated *Ct* exposure. We set out to investigate this hypothesis by returning to the same two provinces of the Solomon Islands six months after MDA took place. This study used tests for two different persistent markers of previous *Ct* infection. The first was an enzyme-linked immunosorbent assay (ELISA) that measured antibodies against the *Ct* antigen Pgp3 ^5, 6^. This tool has been used to assess transmission of both urogenital ^7^ and ocular ^8^ infections, including in the study on Kiritimati Island, where we showed that there were strong associations between *Ct* infection, TF and anti-Pgp3 antibody levels. We also observed a rapid increase in age-specific Pgp3 seroprevalence throughout childhood years in that population ^4^.

This study also assessed trachomatous scarring. Scarring, caused by immuno-pathological responses to repeated cycles of infection, is an irreversible process that, like *Ct* seropositivity, is generally considered to be a persistent marker of previous ocular *Ct* infection. In trachoma, it is characterised by a gradual accumulation of scar tissue in the tarsal conjunctivae ^9^, which typically begins to develop to the point of being visible in late childhood. Scarring is more commonly found in those who have experienced prolonged, severe inflammation and infection ^9– 11^. Very few young children in trachoma-endemic communities have signs of scarring, but as many as 10–30% of older children may do so ^12, 13^. Scarring progresses throughout a lifetime and, in severe cases, is the underlying cause of TT ^11^. Assuming that trachoma was an endemic problem in this population, we would expect to observe an age-dependent accumulation of scarring, with an increasing proportional representation of severe scars with advancing age.

## Methods

### Ethics statement

The methods used in this study adhered to the tenets of the Declaration of Helsinki. Ethical approval for the study was granted by the London School of Hygiene & Tropical Medicine (LSHTM; 8402) and Solomon Islands National Health Research Ethics Committee (HRC15/03). Subjects aged 18 years or older gave written, informed consent to participate. A parent or guardian provided written, informed consent on behalf of those aged under 18 years.

### Study design

To enable comparison to pre-MDA data, only villages in Temotu and Rennell & Bellona provinces where baseline mapping had been conducted were eligible for inclusion. Due to their small respective populations (in the 2009 census, the population of Temotu was 21,362 and of Rennell & Bellona was 3041), the two provinces were combined into one evaluation unit during baseline mapping. The survey took place in June–July 2015, six months after a single round of azithromycin MDA had been delivered by the Solomon Islands National Trachoma Elimination Program.

Thirteen villages were selected in which more than 10% of the community (all ages) had previously had signs of TF ^3^. We included numbers of villages in each province to reflect the proportion of the total population of the two provinces combined (Temotu: 11 villages; Rennell & Bellona: 2 villages). The proportions of active trachoma and infection cases in study villages before MDA were extracted from the full baseline dataset and are presented here for comparison.

This survey was powered to estimate the prevalence of anti- *Ct* antibody seropositivity in children aged 1–9 years. Based on the low prevalence of ocular *Ct* infection prior to MDA (1.3%), we expected the seroprevalence to be approximately 10%, in line with other communities with low *Ct* prevalence ^14^. To estimate seroprevalence with ±5% precision at the 95% confidence level assuming a design effect of 2.65 (as utilised in the baseline study) at least 367 children were required ^15^. In our pre-MDA PBPS survey, we examined a mean of 1.1 children per household and therefore estimated that 25 households in each of 13 clusters were needed to reach our sample size. All residents aged 1 year or above living in households drawn at random from a list of all households in a study cluster were eligible to participate.

### Trachoma grading

Clinical TF, TI and TT grading was performed in the field by two Global Trachoma Mapping Project (GTMP)-certified graders wearing 2.5× binocular magnifying loupes. Graders were trained according to a training scheme developed under the GTMP which was designed to be as standardisable as possible between countries. To become certified, graders were required to achieve a kappa score of ≥0.7 compared to expert consensus on a set of 50 photographs, then a kappa of ≥0.7 compared to a highly experienced grader on 50 schoolchildren’s eyes ^16^. Clinical grading of TF, TI and TT is a routine part of trachoma surveys and prevalence estimates of TF and TT are the basis for programmatic decision making. These signs have, therefore, been the focus of programmatic scale-up of mapping activities whereas similar standardisation does not exist for scar grading. Clinical grading according to the WHO simplified system ^17^ was therefore used for TF, TI and TT, whilst photo-grading was used for scarring.

High-resolution digital photographs of the right tarsal conjunctivae were graded for scarring using the modified WHO trachoma grading system ^18^. Photographs were graded by two photo-graders who had previously achieved kappa scores for inter-grader agreement of >0.7 for F (follicles), >0.7 for P (papillae) and >0.7 for C (conjunctival scar) grades, compared to a highly-experienced trachoma grader. Photographs were graded for F, P and C. The F grades were used to retrospectively check the accuracy of the TF field grading, although it should be noted the two grading systems are not entirely concordant. Photograph grading was undertaken masked to field grading, laboratory results and the other photograph grader’s assessment. Discrepant grades were arbitrated by a third highly experienced grader. Grading was performed using “ FPC_Grader”, an open source software tool based on R.

### Specimens

Dried blood spots were collected for assessment of anti-Pgp3 antibody level. Participants’ fingers were cleaned and then pricked with new, sterile lancets, and blood was collected onto filter paper calibrated to absorb 10 µL (CellLabs, Sydney, Australia). Filter wheels were air-dried for 4–12 hours before being sealed in plastic bags with desiccant sachets. These were refrigerated for up to one week and then stored at -20°C before shipping at ambient temperature to LSHTM, London, UK, where they were again stored at -20°C.

Swabs were passed three times (with a 120°-turn between each pass) over the right conjunctiva of children aged 1–9 years. The examiner and specimen manager took precautions to avoid cross contamination between participants or swabs in the field. In each village, one clean swab was passed within 20 cm of a seated participant and then processed identically to participant swabs to test whether cross contamination between swabs took place in the field. Swabs were refrigerated for up to one week and then stored at -20°C before shipping to LSHTM on dry ice for processing.

### Serological and nucleic acid testing

Anti-Pgp3 antibody level was assessed using ELISA, as described elsewhere ^4, 19^. Optical density (OD) at 450 nm was measured using SpectraMax M3 photometric plate reader (Molecular Devices, Sunnyvale, USA) then normalised to a 20% dilution of high-titre (presumed positive) serum in low-titre (presumed negative) serum.

DNA was extracted from swabs with the QIAamp DNA mini kit (Qiagen, Manchester, UK). Samples were tested for *Homo sapiens* ribonuclease subunit (RPP30; endogenous control) and open reading frame 2 of the *Ct* plasmid (diagnostic target) using a previously evaluated droplet digital PCR assay ^20^ with minor modifications ^21^.

### Data analysis

All data analyses were conducted using R 3.2.3 ^22^. Pre- and post-MDA proportions were compared using Wilcoxon’s rank sum test. Fleiss’ Kappa scores were calculated using the ‘irr’ package in R. ddPCR tests for current ocular *Ct* infection were classified into negative and positive populations according to methods described previously ^20^. Anti-Pgp3 antibody titre was divided into two populations using an expectation-maximisation finite mixture model ^6^, with individuals classified seropositive if their normalised OD was more than three standard deviations above the mean of the presumed-negative population. Using this method, the threshold normalised OD value for positivity was 0.7997. Data from comparable studies in Bijagos Islands, Guinea-Bissau ^23^, and Kiritimati Island, Kiribati ^4^, are shown in Figure 2 and Figure 3, respectively. These have been reproduced under the Creative Commons Attribution 4.0 International ( CC-BY 4.0) and CC BY 3.0 IGO licenses, respectively, to illustrate how patterns of antibodies and scars look in hyper-endemic settings.

## Results

### Study demographics

1511 people (46.3% male; 466 1–9-year-olds) aged 1 year and over were examined in 382 households from the 13 selected study villages. By comparison, the pre-MDA survey of the same villages yielded 1534 people (490 1–9-year-olds) in 394 households. Data on non-participation were not collected in the June 2015 study, but the number enrolled was similar to that for the pre-MDA survey, suggesting a similar participation rate of around 90% on both occasions. In this study, there was a mean of 4 people per household aged 1 year and over, and a mean of 1.2 children per household aged 1–9 years. After accounting for non-participants, this is similar to the means in the 2009 Solomon Islands National Census (4.9 people of any age and 1.4 children aged 1–9 years per household in Temotu, 4.4 people of any age and 1.1 people aged 1–9 years per household in Rennell & Bellona) ^24^.

### Clinical examination for trachoma

Prior to MDA, there were 167/490 (34.0%) cases of TF and 1/490 (0.2%) case of TI in either eye in those aged 1–9 years in study villages ^3^. We observed 66/466 (14.2%) cases of TF and no cases of TI in either eye after MDA, representing a decrease in TF of 58% (p<0.0001). Following MDA, 65% of TF cases were bilateral. Retrospective comparison of field grade of TF to photograph grade of F2–3 indicated moderate agreement between field and photograph grade (kappa = 0.52).

In the two villages enrolled in Rennell & Bellona, a slight increase in the prevalence of TF in either eye in those aged 1–9 years following MDA was noted, but it was not statistically significant (11/60 [17.9%] before MDA to 14/78 [18.3%] after MDA; p=0.956). In contrast, in the 11 enrolled villages of Temotu, a substantial decrease in TF (from 156/430 [36.3%] before MDA to 52/388 [13.4%] after MDA; p<0.0001) was observed ( Table 1).

**Table 1.** Characteristics of study populations before and after MDA, 13 selected communities of Temotu and Rennell & Bellona Provinces, Solomon Islands.

Characteristic	Pre-MDA (October– November 2013 ^3^)	Post-MDA (June–July 2015: this study)	p-value *
Number examined, all ages	1534	1511	-
Number examined aged 1–9 years	490	466	-
Number of households enrolled	394	382	-
% male of those examined	46.5	46.3	0.836
*Ct* infection in those aged 1–9 years **	5/462 (1.1%)	8/457 (1.8%)	0.259
Active trachoma in right (swabbed) eye	160/490 (32.7%)	61/466 (13.1%)	<0.0001
*Ct* infection in those aged 1–9 years with TF **	5/159 (3.1%)	6/61 (9.8%)	0.08
Median *Ct* infection load in positive specimens (plasmid copies/swab)	14,260	18,725	0.175

*Ct: Chlamydia trachomatis*., * Wilcoxon rank sum test. ** Swabs with no detectable human material were not included in this analysis.

No cases of TT were identified during this study.

### Photographic assessment of trachoma

**Figure 1.**
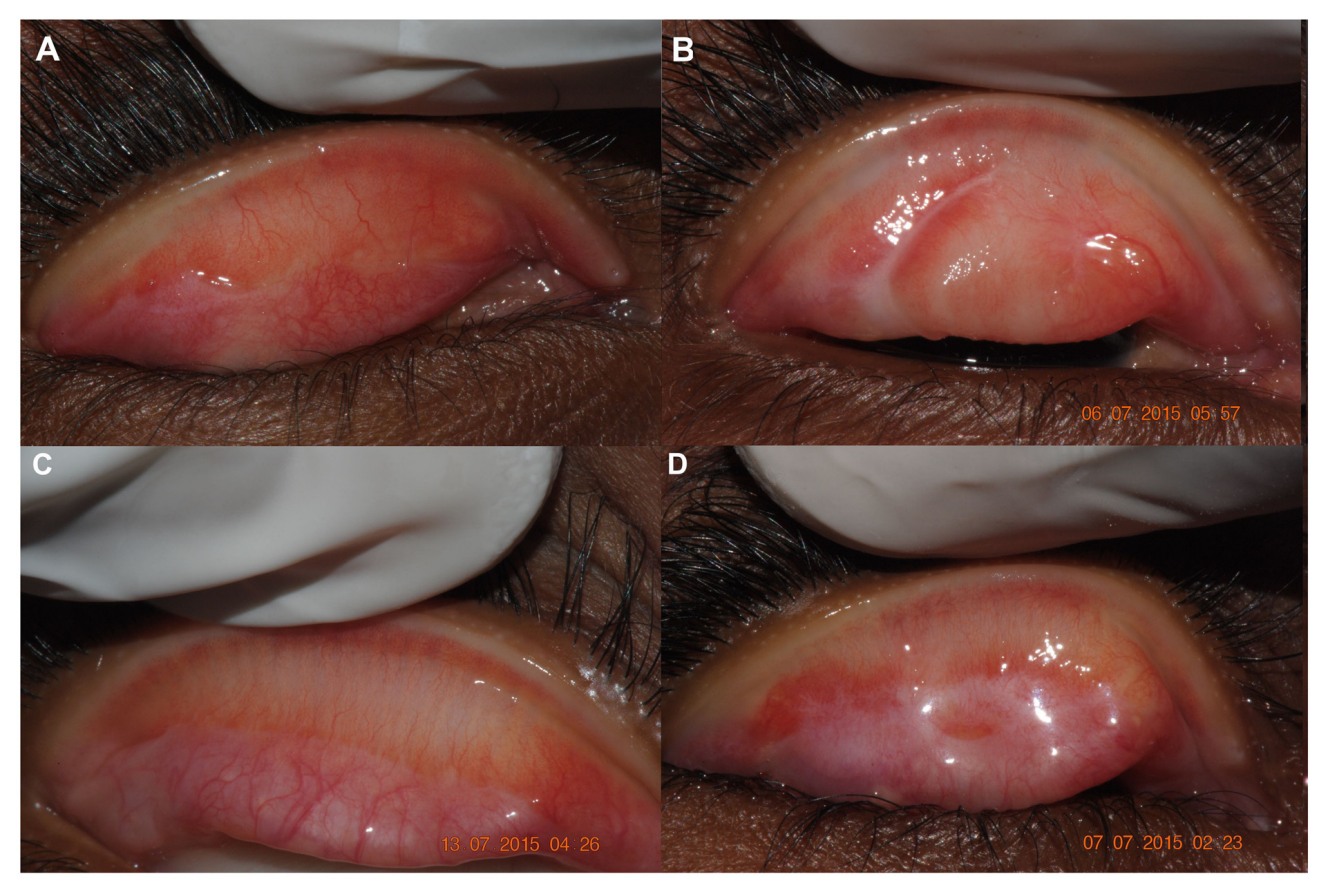
Conjunctival photographs graded as C3 from children aged (clockwise from top left) 7, 6, 1 and 8 years living in Temotu Province, Solomon Islands, June–July 2015. The children in photographs A, C and D are Pgp3 seronegative.

Of the right eye photographs that were collected, 1440/1511 (95.3%) were suitable for grading conjunctival scarring. 42% of photograph grades did not match on either F, P or C grade and so were adjudicated (the majority of these discrepancies were due to disagreements in P grade). 188/1440 (13.1%) photographs were graded as having visible scars (C>0), of which 127 were C1 (mild), 53 were C2 (moderate) and eight were C3 (severe). Four out of eight cases of C3 were found in children aged 1–9 years, these photographs are shown in Figure 1. The photo-graders noted that whilst some conjunctivae met the criteria for classification of C3 (i.e., there was clear scarring with distortion) these photographs also demonstrated the presence of features that are not typically associated with trachoma. In some cases, these were characterised by pronounced linear boundaries between heavily scarred conjunctiva and apparently healthy tissue ( Figure 1C and 1D). Photo-graders noted that 4/53 (7.5%) C2 cases and 3/8 (37.5%) C3 cases looked atypical for trachomatous scarring. Of individuals with eyelid scarring considered typical for trachoma, 36/54 (67%) were seropositive, whereas 2/7 (29%) of those with atypical scarring were seropositive. This difference in proportions was not significant (chi-squared test p=0.123), presumably because of the small numbers with atypical scarring.

**Figure 2.**
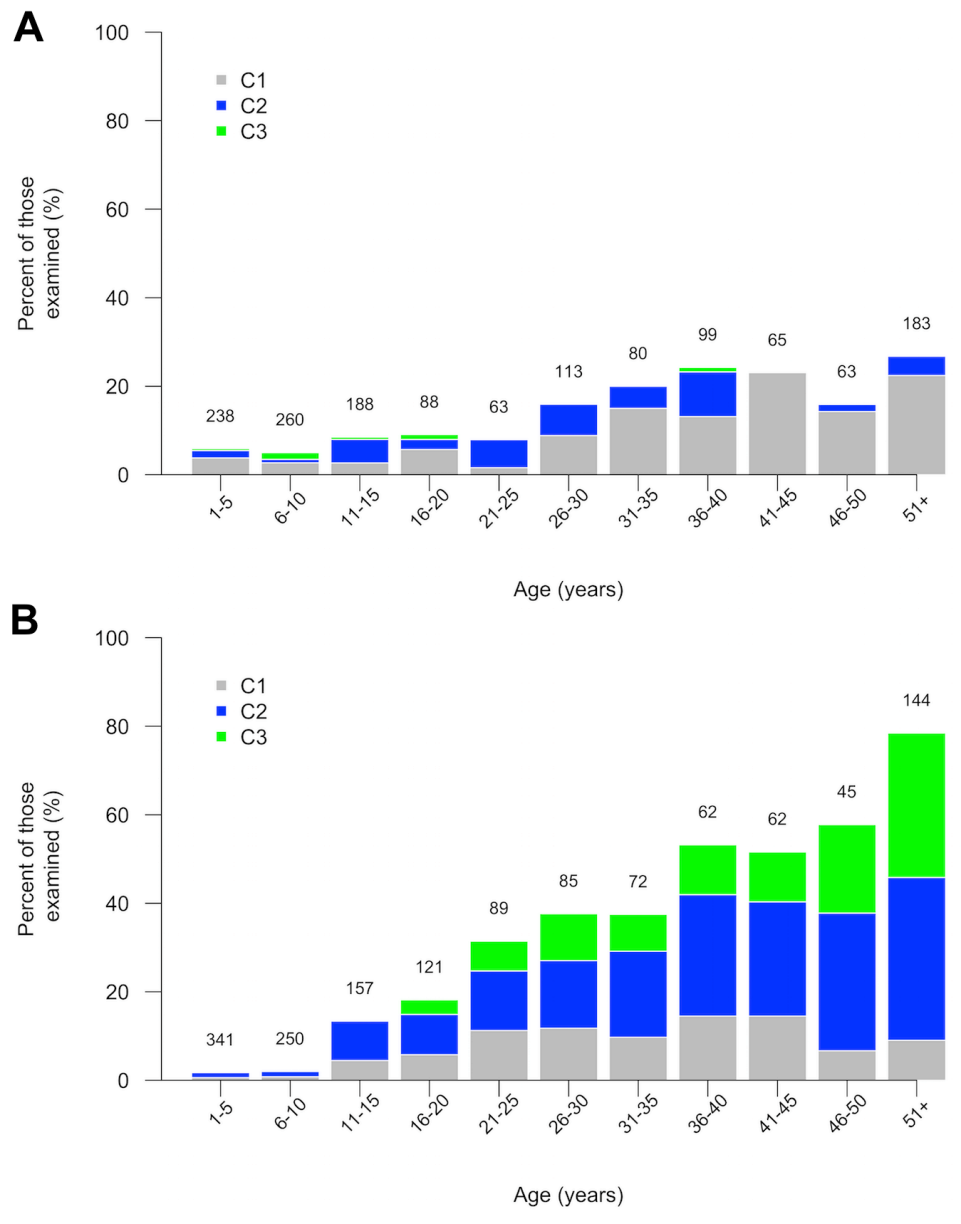
( **A**) Age-specific prevalence of scarring (defined as C > 0), as identified by photograph grading, in 13 selected communities of Temotu and Rennell & Bellona Provinces, Solomon Islands, June–July 2015. ( **B**) Published age-specific prevalence of scarring (defined as C > 0) from a comparator population in Guinea-Bissau, West Africa. Reproduced from Last *et al.* ^23^ under CC-BY 4.0. In both plots, the numbers over each bar represent the size of each group.

**Figure 3.**
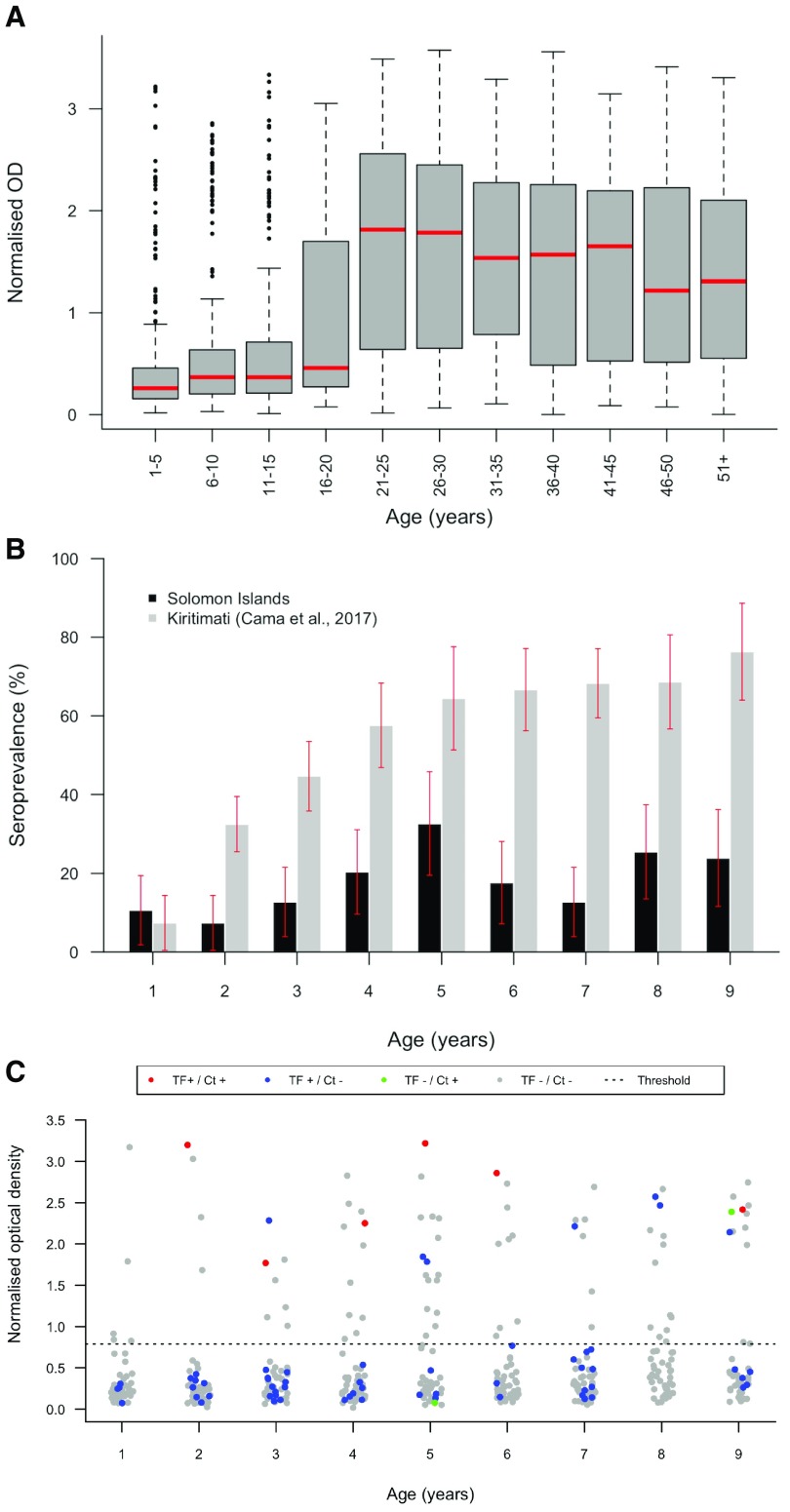
( **A**) Distribution of anti-Pgp3 antibody levels in Solomon Islanders resident in 13 selected communities of Temotu and Rennell & Bellona Provinces, divided into 5-year age groups. Red lines indicate age-group median, grey boxes represent inter-quartile range and whiskers represent the up to 1.5-times the inter-quartile range. For the purposes of this plot, those further than 1.5-times the inter-quartiles range from the upper/lower quartile are treated as outliers, denoted by black spots. ( **B**) Black bars represent age-specific seroprevalence of anti-Pgp3 antibodies from children aged 1–9 years in study communities. Grey bars represent comparator population from Kiritimati Island, Kiribati, surveyed in November 2016 and tested using the same methodology as the Solomon Island samples. Reproduced from Cama *et al.* ^4^ under CC BY 3.0 IGO. Red arrows represent 95% confidence interval of each age-specific seroprevalence estimate. ( **C**) Blood anti-Pgp3 antibody levels from children aged 1–9 years in selected communities.

The age-specific prevalence of scarring in the Solomon Islands is shown in Figure 2A. Of 435 photographs graded from children aged 1–9 years, 25 (5.7%) were graded as C>0. In 311 adults aged >40 years who were examined, 74 (23.8%) had C>0 (65 cases of C1, 9 cases of C2, 0 cases of C3). We have reproduced published data from a comparable study in the Bijagos archipelago, Guinea-Bissau, where ocular *Ct* infections were common (22% of 1–9 year olds had detectable *Ct* infection) ^23^. These data are included to demonstrate the contrast between photo-grading data sets from the Solomon Islands and Guinea-Bissau, the latter of which reflects the typical patterns of scar accumulation that would be expected in a setting where ocular *Ct* infection is hyperendemic ( Figure 2B).

In the Solomon Islands, the proportion of people with C1 increased with age (logistic regression p<0.0001), but the proportion of people with more severe scarring (C2 or C3) did not (logistic regression p=0.149). There was also no significant association between having C>0 and gender (chi-squared test p=0.80). In Rennell & Bellona, 25/225 (11.1%) of photos were graded C>0, whereas in Temotu, 163/1215 (13.4%) of photos were graded C>0; the difference in scarring between provinces was not significant (chi-squared test p = 0.404).

### Anti-Pgp3 serology

Dried blood spots were collected from 1499/1511 (99.2%) people aged ≥1 year during the post-MDA survey; the other 12 people declined finger-prick. The distribution of normalised OD for all individuals, grouped into five-year age brackets, is shown in Figure 3A. This figure demonstrates the median normalised OD to be much higher in people aged >25 years than their younger counterparts. Overall, 633/1499 (42.2%) people were classified as seropositive. In children aged 1–9 years, the prevalence of anti-Pgp3 antibodies was 83/462 (18.0%). In 1-year-olds alone, it was 5/47 (10.6%). The mean seroprevalence in those aged 6–10 years was not significantly higher than in those aged 1–5 years (20.3% compared to 16.6%, chi-squared test p = 0.328) ( Figure 3C). In Figure 3B, we have also included comparator data from Kiritimati Island, where the TF prevalence was similar but where the prevalence of *Ct* infection was much higher ^4^. Among children aged 1–9 years, the rate and dynamics of accumulation of seropositivity differed substantially between the Solomon Islands and Kiritimati ( Figure 3B).

The largest increase in seroprevalence was observed between those aged 16–20 years and 21–25 years where seroprevalence rose significantly from 30.4% to 71.6% (chi-squared test p<0.0001). Of those aged over 25 years, 67.4% were seropositive. In the 16–20-year-old age group, the prevalence of seropositivity amongst females was higher than in males (41.1% versus 13.9%, chi-squared test p<0.0001). The seroprevalence among children in Rennell & Bellona was significantly higher than that in Temotu (38.5% versus 13.8%; chi-squared test p<0.0001).

80.3% of Solomon Islands children with TF were seronegative. 82.3% percent of children without TF were seronegative. There was no association between seropositivity and signs of TF in children aged 1–9 years (logistic regression adjusted for age and gender p=0.616). In those who were younger than the population median self-reported age of sexual debut (18 years ^25^) there was no association between C grade and anti-Pgp3 OD (linear regression adjusted for age and gender p=0.453) or anti-Pgp3 positivity (logistic regression adjusted for age and gender p=0.472).

### Ocular *C. trachomatis* infection

Positive endogenous control tests were obtained from 457/466 swabs from children aged 1–9 years. The median load of the endogenous human RPP30 target was 33,500 copies, equivalent to over 15,000 conjunctival cells. In this study, 8/457 (1.8%) children had evidence of *Ct* plasmid DNA. Of the eight specimens from children who were positive for *Ct,* the median load was high at 18,725 plasmid copies/swab. This suggests that these were much less likely to be false positive results than had they been low load infections. ** 6 (9.8%) of the 61 children with TF also had *Ct* infection. We previously showed that, of 462 swabs from the pre-MDA study which passed quality control, 5/462 (1.1%) had infection. All five infection cases came from children with active trachoma in the right eye (5/159, 3.1%) *.* The median pre-MDA load of *Ct* infections in those villages was 14,260 plasmid copies/swab ^3^. Neither the difference between the pre- and post-MDA *Ct* prevalence nor the pre- and post-MDA *Ct* load were statistically significant (Wilcoxon rank sum test p=0.259 and p=0.175, respectively). The relationship between *Ct* infection, signs of trachoma and seropositivity was examined in children aged 1–9 years and is summarised in Table 2. 7/8 cases of infection were in seropositive individuals ( Figure 3C). All study villages had at least one case of TF, but infections were limited to five of the 13 villages studied. Two villages in Rennell & Bellona were the sites of five of the eight *Ct* infections identified during this study.

**Table 2.** Serological status compared to other tests for trachoma, 13 selected communities of Temotu and Rennell & Bellona Provinces, Solomon Islands, June–July 2015.

Comparator		1–9 year-olds			≥10 year-olds		
		Seronegative	Seropositive	Total	Seronegative	Seropositive	Total
*Ct* infection by ddPCR *	Positive	1	7	8	-	-	-
	Negative	373	76	449	-	-	-
TF	Positive	53	13	66	13	9	22
	Negative	326	70	396	474	541	1015
Scarring	C0	333	77	410	414	418	832
	C1	15	1	16	36	75	111
	C2	3	2	5	16	32	48
	C3	3	1	4	1	3	4

*Ct: Chlamydia trachomatis;* ddPCR: droplet digital polymerase chain reaction; TF: trachomatous inflammation—follicular. * Swabs with no detectable human material were not included in this analysis.

## Discussion

Based on moderate estimates of province-level prevalence of TF, the Solomon Islands has (along with other Pacific Island states) been identified as having endemic trachoma. Whilst measures for trachoma elimination have already been deployed in Temotu and Rennell & Bellona, we have previously noted that TI, ocular *Ct* infection and late-stage disease (TT) are rare ^3^. If the village-level findings of the current study were replicated throughout the district, then TF would still be sufficiently prevalent to warrant continued intervention. The conjunctival scarring and serological data presented here, combined with previous *Ct* infection data, suggest that ocular *Ct* is scarce and is not being widely transmitted. TF is not concurrent with an appreciable burden of infection, severe scarring or TT in this population. Our most significant finding, which is that 80% of TF cases occur in people who are seronegative for antibodies against *Ct*, questions whether further rounds of MDA are warranted in this population.

In Kiritimati Island, we found that just 20.3% (23/119) of children with TF were seronegative according to the same ELISA test that was used here. We were not surprised to find some individuals with TF were seronegative because (1) a proportion of individuals who have primary infections will not yet have seroconverted and (2) anti-Pgp3 antibody responses may not be the same in all people due to natural variability in host responses. However, the fact that 80% of TF cases in Solomon Islands were seronegative suggests that many cases are not caused by *Ct* infection. More important still is that in Kiritimati Island, children with TF were far more likely to be seropositive than those without TF. In the Solomon Islands, however, we not only found that most TF cases were seronegative, but that individuals with TF were no more likely to be seroreactive to Pgp3 ** than their peers without TF. We can rule out the possibility that Solomon Islanders are collectively non-responsive to Pgp3 (for genetic reasons, for example) because the majority of the adult population do have antibodies against Pgp3. The most parsimonious explanation of our findings is that TF in this population is caused by a factor other than *Ct*.

We found a small and non-significant increase in age-specific seroprevalence between young children (1–5 years) and older children (6–10 years), which suggests that children here occasionally do encounter *Ct* infections. This is concordant with our previous data, which suggested that although ocular *Ct* strains are present in the Solomon Islands, they are rare ^3^. This contrasts with the data from Kiritimati Island, where we saw that there was a substantial year-on-year increase in age-specific seropositivity ( Figure 3B). The increase in seropositivity with age in this group was also very modest compared with that seen in hyper-endemic villages in Tanzania, where seropositivity has been observed to increase from approximately 25% to 94% between the ages of 1 and 6 years ^26^. In the current dataset, there was a rapid increase in age-specific seroprevalence around the age of 18 years, the self-reported median age of sexual debut in a nearby population ^25^. The prevalence of urogenital *Ct* infection is known to be high in women attending antenatal clinics in the Solomon Islands ^25^, which probably explains the high seroprevalence in adults as anti-Pgp3 antibodies do not distinguish between biovars. Exposure during parturition may also be a major contributor to the 10% of 1-year-olds in our study who had evidence of prior Pgp3 exposure ^27^.

While seroreversion due to clearance of infection by MDA is a possible explanation for the low seroprevalence and absence of association of anti-Pgp3 antibodies with TF in the Solomon Islands, there is currently no evidence for complete seroreversion for Pgp3-specific antibodies ^7, 26^ after clearance of infection. It is, in any case, hard to imagine a biologically plausible situation in which seroreversion would fully account for the contrasts between serological data from Kiritimati and data from the Solomon Islands.

Whilst the proportion of people with mild scars increased with age, the proportion of those with more extensive or eyelid-distorting scars did not increase with age. Contrary to what might be expected in a trachoma-endemic community ^13^, no eyelid-distorting scars were found in 311 adults aged above 40 years. Some cases of severe scarring we observed in children were not typical of trachoma and were found in children who lacked Pgp3 reactivity ( Figure 1, Table 2). There are other inflammatory conditions (e.g. adenoviral, acute haemorrhagic or membranous conjunctivitis) that may result in conjunctival scarring, although the pathology, incidence and prevalence of these are poorly understood and incompletely described ^28^. It is also unclear whether the TF that we observed is directly linked to conjunctival scarring in this setting. In Temotu and Rennell & Bellona, the low prevalence of severe scars suggests that the proportion of the population at risk of developing TT is very low, although we cannot determine whether this might change in the future.

One limitation of all studies of tarsal conjunctival scarring is an element of diagnostic uncertainty when the scarring is very mild; determining whether it meets the C1 criteria is difficult. It is possible that this may be easier to judge from high quality digital photographs than during live examination using 2.5× loupes. In contrast, eyelid distortion, required to meet the criteria for C3, may be difficult to judge from a single, two-dimensional photograph. This can lead to disagreement between graders. However, subjectivity in determining the presence or absence of eyelid distortion is also present with field grading. Photography has the advantage that the images can be presented as empirical evidence ( Figure 1) that can be scrutinised, appraised and regraded by third parties where field grades cannot. Photograph grading is the method of choice for studies of scarring severity or progression ^12, 13, 29^.

The data collected on infection, antibody, scarring and trichiasis prevalence are consistent in their suggestion that trachoma is uncommon in this population despite the moderate TF prevalence. The field TF grades were therefore compared to photograph grades of F2 or F3, yielding a kappa agreement score of 0.52 (moderate agreement). This was considered to be acceptable as this is a post-treatment scenario where there are likely to be more borderline cases where disagreement may be more common. Also, the discrepancy between F2 and TF grades (for example, someone with five follicles in the central conjunctiva would qualify as having TF but not F2) may lead to further disagreement. The most common type of disagreement was field TF+ / photograph F≤1, as we might expect given the discrepancies between the FPC and the simplified system. There is also a possibility that the grader had overcalled some milder cases of follicular inflammation as TF. When a discrepancy occurs, we consider the field grade to be more likely to be accurate as the field grader can achieve a three-dimensional real-time view of the eyelid.

The 13 communities included here were the most highly endemic of those surveyed in Temotu and Rennell & Bellona during the GTMP, with at least 15% of children aged 1–9 years living in selected villages having TF before MDA. In this study, we showed that the burden of TF in many of these villages dropped significantly following a single round of MDA, but still remained above the threshold for continued intervention. The drop in clinical disease was not reflected by a simultaneous drop in ocular *Ct* in children with TF, which actually increased slightly (although this increase was not statistically significant). From interventions in other settings, we might expect TF prevalence to have approximately halved six months after a single round of MDA, given 80% population coverage ^30, 31^. Azithromycin has anti-inflammatory and broad-spectrum antibiotic effects, which may help explain the observed decrease in clinical disease, but would of course only be effective in controlling a subset of bacterial genera. Given the method of village selection, regression to the mean would be another potential explanation for the fall in prevalence.

We observed regional variation across the study villages. Compared to Temotu, we noted that MDA did not have as significant an impact on TF prevalence in Rennell & Bellona, that there were more children there who were seropositive and that there were more children with TF who also had infection. Our survey was not prospectively designed to assess these differences, and the subgroup size in Rennell & Bellona precluded more detailed analysis. Temotu is more similar to the rest of the Solomon Islands in terms of the geology and geography of the islands as well as in relation to the lifestyle and ethnicity of the majority of the inhabitants. Further studies on the localisation of trachoma in the islands are warranted.

The complex, multistage nature of trachoma makes it difficult to predict the outcome of any given intervention ^32^. Data from cross-sectional surveillance tools used in isolation can be hard to interpret, especially given the prolonged persistence of TF after clearance of infection ^33^. Some features of conjunctivitis in the Solomon Islands resemble trachoma, particularly the prevalent follicular inflammation and some of the severe conjunctival scarring. Crucially, we found that these clinical features were not co-endemic with TT at a prevalence that indicated an ongoing public health problem. In this setting, we believe that tests for infection gave a better indication of the public health threat from trachoma than TF. A combined approach in which various age-specific markers of trachoma are assessed together across the complete age range of the population may prove useful for prioritising areas for intervention where the prevalence of TF alone does not coherently reflect trachoma’s public health importance.

Contrary to the WHO recommendation for treatment based solely on prevalence of TF, our data suggest that trachoma is not a public health problem in these villages. Whilst there have been substantial collateral benefits to local residents from having received MDA (such as on genital *Ct* ^34^ and yaws ^35^), further rounds of azithromycin MDA do not appear to be indicated for the purposes of trachoma elimination. As the positive predictive value of TF for ocular *Ct* infection decreases globally in response to declining prevalence, it is likely that other regions and countries will be identified where a high prevalence of TF is not reflective of threat to vision and may not require MDA. WHO recommendations for implementation of MDA and the SAFE strategy should be reviewed in the light of this evidence.

## Data and software availability

Raw data are available from: https://doi.org/10.17037/DATA.279 ^36^

Data are available under the terms of the Creative Commons Attribution 3.0 license (CC-BY 3.0).

FPC_GRADER is available from: https://github.com/chrissyhroberts/FPC_GRADER/

Archived source code as at time of publication: http://doi.org/10.5281/zenodo.1116799 ^37^

License: GNU
