# Supplementary material for: Clinical signs of trachoma are prevalent among Solomon Islanders who have no persistent markers of prior infection with Chlamydia trachomatis
Source: Wellcome Open Res. 2018 Aug 10;3:14. Originally published 2018 Feb 22. [Version 2] doi: 10.12688/wellcomeopenres.13423.2 (PMC5854984; doi:10.12688/wellcomeopenres.13423.2)
Supplement: Peer review history

# Peer review: Referee response for version 1

**DOI:** 10.21956/wellcomeopenres.14575.r31070

**Published:** 2018-03-14

**Authors:** Meraf A. Wolle

**Affiliations:** 1 Dana Center for Preventive Ophthalmology, Wilmer Eye Institute, Johns Hopkins University School of Medicine, Baltimore, MD, USA

This manuscript describes trachoma prevalence in the Solomon Islands over an almost 2 year period. The data highlight the discrepancy between the presence of high TF rates, which would indicate that trachoma is a public health problem in this area, while the rates of other signs of trachoma (ie. TI, TT) are very low to none. The study is well designed and the manuscript well written. The concern raised by the authors that the high TF rates seen may not actually reflect ongoing trachoma infection and thus MDA may not be warranted are well supported by the data as is their suggestion that consideration be given to using multiple markers of trachoma to determine whether or not intervention is needed.

 I have some comments for the authors which I list below:

 TS

1. In the Methods section the authors state that grading of TF, TI, and TT was done clinically and that only scarring was graded by photographs. However, when they describe the inter observer agreement for the grading they refer to the grading of F, P, and C (as per Dawson et al.). Could the authors please report the following:
  - What the inter observer agreement for grading scarring alone was in this paper?
  - What percent of grades had to be adjudicated?
  - Where else this Dawson et. al scarring grading scheme applied to photographs has been used/validated?
2. The authors followed the grading system of conjunctival scarring described by Dawson et al.; the grade descriptions do not have clear cut offs. How did the authors modify this grading system for the photographic graders? How did they determine the cut off between C1 and C2? How did they determine shortening/distortion for C3 from photographs alone? Did they include looking for signs of lid margin conjunctivilization as they state in their C3 example photograph? These are areas that could lead to a great deal of disagreement between graders...
3. In the comparison population whose scarring data is presented in Figure 2 B, was the grading done in the same way as in this paper, through photographs with two graders and an adjudicator? If so, what was the inter-observer agreement? This information was not readily available in the paper cited.

TF

1. Since the authors mention the kappa of F, P, and C, I wonder if they happened to grade follicles and papillae photographically? If so, how does this compare to the field grades? Given the discrepancy between TF and all other signs of trachoma, having a way to ensure accurate field grading of TF would be reassuring.
2. Do the authors have any hypotheses as to why the prevalence of TF might be so high in this community if it is not due to trachoma (ie. higher incidence of viral etiologies, etc.)?

 

 PGP3 Antibody

1. In the discussion the authors state that there is no evidence for complete seroreversion. Although I agree with the overall conclusion that seroreversion cannot account for the differences in the data that were observed, the authors may want to consider looking at/adding the following reference which does support the presence of seroreversion: West et al. (2018) ^1^.

I have read this submission. I believe that I have an appropriate level of expertise to confirm that it is of an acceptable scientific standard.

## References

[1] WestSKMunozBKaurHDizeLMkochaHGaydosCAQuinnTC: Longitudinal change in the serology of antibodies to Chlamydia trachomatis pgp3 in children residing in a trachoma area. *Sci Rep*.2018;8(1) : 10.1038/s41598-018-21127-0 3520 10.1038/s41598-018-21127-0 29476106PMC5824943

# Response

**Published:** 2018-07-13

**Authors:** Robert Butcher

**Affiliations:** London School of Hygiene & Tropical Medicine, UK

We are grateful to Dr Wolle for her feedback on our article. We have taken her comments into account as we prepared the second version of this manuscript, and believe they have enhanced the quality and transparency of the article. We hope the following additional information addresses some of her comments directly.

 The photograph graders had to achieve a kappa score of >0.7 in C grading, a kappa score of >0.7 in F grading and a kappa score of >0.7 in P scoring when compared to a highly experienced grader before they were considered to be ‘validated’ for photograding. It is hard to calculate a kappa score between graders in this particular manuscript because those where the primary graders did not reach consensus were arbitrated. Where the first two grades agreed, the grade was accepted as the final grade. Where the first two grades disagreed on F, P or C, an independent expert adjudicated. There are, therefore, a subset of photographs where both primary graders have called the same C grade, and the arbitrator has also graded C. In these cases, the kappa agreement was 0.76 between primary and arbitrator grades.

 42% of participant photographs did not agree on F or P or C score, so were arbitrated. The majority of these were due to disagreement in P score.

 We have presented data in the article from a comparable study in Guinea Bissau where the same grading process was used, and a starkly different picture emerges. In addition, we have listed below some examples of recent studies using the FPC system.

- Ramadhani *et al.* Immunofibrogenic Gene Expression Patterns in Tanzanian Children with Ocular Chlamydia trachomatis Infection, Active Trachoma and Scarring: Baseline Results of a 4-Year Longitudinal Study. *Front. Cell. Infect. Microbiol.*, 2017
- Zhou *et al. *The conjunctival microbiome in health and trachomatous disease: A case control study. *Genome Medicine*, 2014.
- Roberts *et al.*, Conjunctival Scarring in Trachoma Is Associated with the HLA-C Ligand of KIR and Is Exacerbated by Heterozygosity at KIR2DL2/KIR2DL3. *PLoS NTDs*, 2014.

While we are aware of other grading systems developed specifically for grading scarring in photographs, we considered this grading system to be the best balance between the relatively simplistic tool of the simplified system and the highly complex tools described by others. We felt the experience our group had using the FPC system would lead to more reproducible grading which would outweigh the benefit incurred by using more specialised methods. We acknowledge that the difference between borderline cases of C0 and C1 or between C1 and C2 may be difficult to arbitrate, although felt that was a problem common to all trachoma grading, for example with regards to interpretation of what constitutes a follicle >0.5mm or the difference between different levels of inflammation. Regarding the question of distortion of the tarsus, we believe in the photographs where C3 was called (examples of which are displayed in figure 1) the distortion of the tarsus is clear, however, it is possible that some cases of C3 were missed. We believe at the population level, this is unlikely to lead to gross shifts in results, especially in the context of this population where over 85% of people show no scarring at all. Finally, the comparator data we present in figure 2b demonstrate that the same grading scheme used in an area with hyperendemic infection does identify cases of moderate and severe scarring.

 In the comparator population from Guinea Bissau, the photographs were reviewed by two experienced graders who met to discuss the outcome of discrepant results. Further information about this process was not recorded, so it is not possible to determine what proportion needed to be discussed.

 The kappa agreement score between photograde of F2–3 and field grade of TF is 0.52 and additional information surrounding this comparison is included in response to Dr Nash’s comment.

 The two hypotheses that have emerged as a result of this set of work are (1) that an as-yet uncharacterised, non-bacterial cause of follicular conjunctivitis is inflating estimates of TF or (2) that the host response of residents of the Solomon Islands to circulating *Ct *infection is not the same as that in other countries, so does not result in the antibody and cicatrizing responses seen in other endemic areas. We consider the former to be more likely, and mention that in the final sentence of the second paragraph in the discussion.

 Thank you for directing us to the paper from West *et al.*. Our interpretation of the data in that paper is that the small proportion of people classed as sero-reverters at one year actually have a distinguishably lower antibody level at baseline than those who were positive at both timepoints, and a distinguishably higher antibody level at follow up than those classed as negative at both timepoints, therefore we believe there is no evidence for ‘complete’ seroreversion, and the statement should stand.

# Peer review: Referee response for version 1

**DOI:** 10.21956/wellcomeopenres.14575.r31073

**Published:** 2018-03-12

**Authors:** Scott D. Nash

**Affiliations:** 1 Trachoma Control Program, Carter Center, Atlanta, GA, USA

The manuscript describes the results of a trachoma prevalence survey which measured clinical signs, chlamydial infection, as well as anti-Pgp3 seropositivity among a population from the Solomon providences of Temotu, and Rennell & Bellona. This report is one of a number of recent reports which have examined this population in great detail, including through the use of conjunctival transcriptome profiling and measures of microbial diversity. The manuscript is well written and the laboratory and analytic techniques were appropriate for the main aims of the survey. Within this population, at both baseline, and at the survey time-point described in this manuscript, the clinical signs of trachoma were high enough to warrant interventions with antibiotics per WHO guidelines. However, other markers of trachoma, such as low infection and seropositivitiy, as well as the very low prevalence of trichiasis, suggested the possibility of alternative etiologies for the clinical signs (TF) of trachoma in these providences. The findings of this study are important, as is the author’s call for the use multiple age-specific markers of trachoma to help in prioritizing areas for intervention.

 Major Comments

- Given that a major finding here is that TF remained above 10%, while other trachoma indicators suggested that trachoma was not a public health problem in these providences, it would strengthen the manuscript if the authors provided some more information on whether or not they took additional steps to ensure accurate grading of TF in the field. For example:
- The authors state that one of two GTMP-certified graders did the grading for TF, TI, and TT. Please state the month and year that this individual was certified for this particular survey, and what criteria were used for certification. The kappas achieved were reported for the photograph grading, please report for the field grading.
- Second, it appears that this study used only one grader to capture field grades. Is there a concern that the single chosen grader may have been a person who systematically overcalls TF (more likely to call borderline cases TF)?
- Were there any quality control steps taken to ensure the quality of the field TF grading? Given that the discrepancy between TF and other markers of trachoma were discovered in these providences at an earlier timepoint (Butcher et al 2016), and that this current survey for these providences was already more thorough than standard trachoma surveys, the authors could have taken some steps to ensure the quality of field grading. For example, was it possible to recheck a certain percentage of TF cases while still in the field; how did the photo graders compare to the field graders for the TF grade within this particular survey?

 

 Minor comments

- Under trachoma grading in the methods section, the authors discuss standardization of clinical signs but only cite Thylefors in the section. That paper shows comparable inter- and intra-observer variation for TF, TI and scarring (TS). Is there a different reference that can be used to support the assertion that TF, TI, and TT are more “standardized” than scar grading?

I have read this submission. I believe that I have an appropriate level of expertise to confirm that it is of an acceptable scientific standard.

# Response

**Published:** 2018-07-13

**Authors:** Robert Butcher

**Affiliations:** London School of Hygiene & Tropical Medicine, UK

We appreciate the time Dr Nash has taken to appraise this manuscript. We include a point-by-point response to his comments below. We believe his feedback has improved the article, and hope he agrees.

 Graders were trained according to the GTMP training protocol as described elsewhere. The training was robust; trainees attended a taught course on trachoma, were required to achieve a kappa score of ≥0.7 when grading a set of 50 photographs in which the trachoma grade had been agreed by several experts and were required to achieve a kappa score of ≥0.7 against a highly experienced grader trainer in the assessment of 50 children aged 1–9 years in the field. Both graders working on this study had met those criteria on the first attempt during a GTMP training in the Solomon Islands in September 2013 (unfortunately we do not have their specific kappa scores available). The grading in Temotu (where 11 of the 13 study villages were located) was undertaken by a single grader who had also successfully completed a GTMP training course in Ethiopia prior to the Solomon Islands course. A second grader conducted the grading in Rennell & Bellona (2 of the 13 study villages were in this province). We believe by training in this manner, we have ensured our data is as comparable as possible to data generated by the GTMP, and by impact and pre-surveillance surveys in the majority of trachoma-endemic areas. Data generated by graders trained in this manner are used to guide treatment decisions around the world. However, to support our research programme alongside the Solomon Islands National Trachoma Control programme we have collected photographs to enable us to retrospectively check grading. In a sample of photographs from a previous survey, the kappa score between field grade of TF and photograde of TF was 0.88. In this survey the photographs were graded with the FPC system rather than the simplified system. The kappa agreement between a photograph grade of F2/F3 and field grade of TF was 0.52, indicating moderate agreement. This is lower than in previous studies in the Solomon Islands, potentially because of more borderline cases in a post-treatment community or because F2/F3 is not exactly comparable to TF. We have expanded the methods section in version 2 to give a fuller account of the training process, we have added the F2/F3 vs TF kappa score to the results, and have added a section in the discussion regarding this process.

 Two graders were involved in this study. Both had met the predefined criteria for successful grading and were therefore accredited to grade trachoma to the quality required to guide treatment decisions. The kappa agreement between field and photograph grades along with caveats about expectations for that agreement has been included in the manuscript in line with the previous comment. The most common type of disagreement was field+ / photograph-, therefore there is a possibility that that grader had overcalled some milder cases of follicular inflammation as TF. For the reasons described above, we expect a certain number of discrepancies. Also, when a discrepancy occurs, we believe the field grade to be more likely to be accurate as the field grader can achieve a three-dimensional real-time view of the eyelid. It is worth noting that the key finding of this paper that the majority of TF cases do not have antibodies to Pgp3 holds true if we define the clinical phenotype using the photograph grades (70% of children whose photographs were graded as F2/3 were Pgp3 seronegative, chi-squared test of association p = 0.49).

 It wasn't possible to recheck grading in the field, however, we have presented the comparisons between field and photograph grade above and in the paper.

 Because specific interventions at the programmes are guided by TF, TI and TT grades but not TS grades, scale up of mapping activities has focussed on those signs. Therefore, grading for TF, TI and TT has been repeatedly replicated in thousands of districts round the world, whereas TS grading is relatively uncommon. Therefore, while graders may be able to be trained to assess TS reliably, training for TF, TI and TT is ‘standardised’. We have amended the terminology in the methods to reflect this, and added a reference where this is also discussed.
